# Alice in Wonderland Syndrome in dementia with Lewy bodies: a case report exploring visual cognition dysfunction

**DOI:** 10.3389/fneur.2025.1556218

**Published:** 2025-03-27

**Authors:** Alexis Demas

**Affiliations:** Hospital Group Du Havre, Le Havre, France

**Keywords:** Alice in Wonderland Syndrome, Lewy body dementia, visual cognition, visual hallucination, neuroscience

## Abstract

**Background:**

Alice in Wonderland Syndrome (AIWS) is characterized by transient distortions in visual perception—alterations in size, shape, and spatial relationships—typically described in migraine or encephalitis. Its occurrence in neurodegenerative conditions, particularly in dementia with Lewy bodies (DLB), remains exceedingly rare.

**Case description:**

This article reports a case of a 68-year-old patient with dementia with Lewy bodies (DLB; limbic-early subtype) who presented with typical DLB features alongside a brief episode of misperception—reporting that his bed had “shrunk.” Neuroimaging revealed diffuse cortical atrophy with prominent bi-hippocampal and parietal lobe involvement, and hypoperfusion on HMPAO SPECT.

**Conclusion:**

This is the first reported case of AIWS in a patient with DLB. We hypothesize that selective dysfunction of high-level visual networks—particularly in the right extrastriate cortex responsible for the canonical storage of object size—may lead to an agnosia of object size. This case underscores the importance of considering AIWS within the spectrum of visual disturbances in DLB.

**Theoretical implications:**

These findings provide novel insights into the neurobiology of visual cognition, aligning with Husserl’s concept of the “primordial body” (Urleib) and intuition. They suggest that disruptions in the integration of visual sensory inputs and canonical object properties may critically influence the conscious reconstruction of reality.

## Introduction

In an era of rapid technological advances and emerging neurotechnologies, our understanding of the brain’s cognitive and perceptual mechanisms is continually evolving. Recent developments—such as brain implants that decode neuronal activity—have deepened our insights into the neural basis of perception and cognition (1, 2). These advances, however, do not negate the uniquely human experience of perception and its subjective reconstruction into thought—a process that eludes artificial intelligence and remains deeply intertwined with our personal history and embodiment.

Phenomenological approaches, notably those of Edmund Husserl, emphasize the “primordial body” (Urleib) and intuition as foundational to human experience (3, 4). Husserl’s work reminds us that our perception of reality is not a mere sensory registration but a synthesis influenced by memory, emotion, and prior experience (5, 6). This concept resonates with clinical observations in neurology, where disruptions in perceptual integration—such as those seen in split-brain patients (7)—shed light on the role of interhemispheric transmission via the corpus callosum.

Visual cognition is a complex process. It involves the initial acquisition of visual information—such as color, volume, size, speed, and movement—followed by its storage and subsequent comparison with stored canonical knowledge (8). This process, which engages the hippocampi, limbic system, parietal, temporal, and frontal lobes, and various subcortical structures (9), is essential for constructing our mental image of reality. Although it is often stated that approximately 25% of the brain is devoted to processing visual information (10), such claims must be supported by current literature. Moreover, the clinical presentation of Lewy body dementia (LBD) is heterogeneous; its subtypes vary according to the initial site of proteinopathy (olfactory, limbic, or brainstem) and its subsequent propagation (11, 12). While visual hallucinations and illusions are well recognized in LBD, the occurrence of Alice in Wonderland Syndrome (AIWS) within this context has not been previously reported.

AIWS is a rare disorder—characterized by metamorphopsias, altered body schema, and distorted time perception—that has primarily been associated with migraine and viral encephalitis (13, 14). The present case report describes a patient with DLB exhibiting a brief, yet distinct, visual misperception (a reported “shrinking” bed).

This work focuses on describing Alice in Wonderland Syndrome in a patient with Lewy body dementia (limbic-early subtype). This is the first description of this syndrome in this entity, but more importantly, it updates its phenomenology. An agnosia of object size should thus be considered, linked to the impairment of high-level neuronal networks located in the extrastriate cortex of the right occipital lobe (metacognition) involved in controlling the analysis of the canonical form of objects perceived by the visual system. Moreover, these networks could also constitute the stage of visual cognition allowing the elaboration of the mental image intended to coherently reproduce the visual scene seen, which will subsequently be processed by other cognitive processes (language, executive functions), thereby providing a unique insight into the mechanisms by which the brain transforms reality into thought.

## Case report

A 68-year-old patient was transferred to the Neurology department due to falls and a confusional syndrome. The only significant medical history was hypertension. There was no family history of neurodegenerative diseases. A recent visual examination revealed mild bilateral presbyopia (no associated retinopathy). The family reported progressive neuropsychiatric symptoms over the past year, including apathy, anxiety, and delusional thoughts (paranoia, persecution). There was also a noted decline in cognitive performance (attention, memory, language), varying in intensity daily. The patient’s autonomy was deteriorating. He wandered, was sometimes aggressive, and had an inverted sleep–wake cycle. Neuroleptic treatments (Haldol, Risperdal) were initiated. Subsequently, the patient exhibited a marked deterioration with falls and a confusional syndrome leading to hospitalization. Upon admission, the patient was alert but slow, with incoherent speech. Visual hallucinations were suspected (staring and attempting to grasp non-existent objects), which were not highly distressing. A severe akinetic-rigid parkinsonian syndrome, bilateral (slightly asymmetric, predominating on the right side) with a significant axial component, was noted.

**Figure 1:**
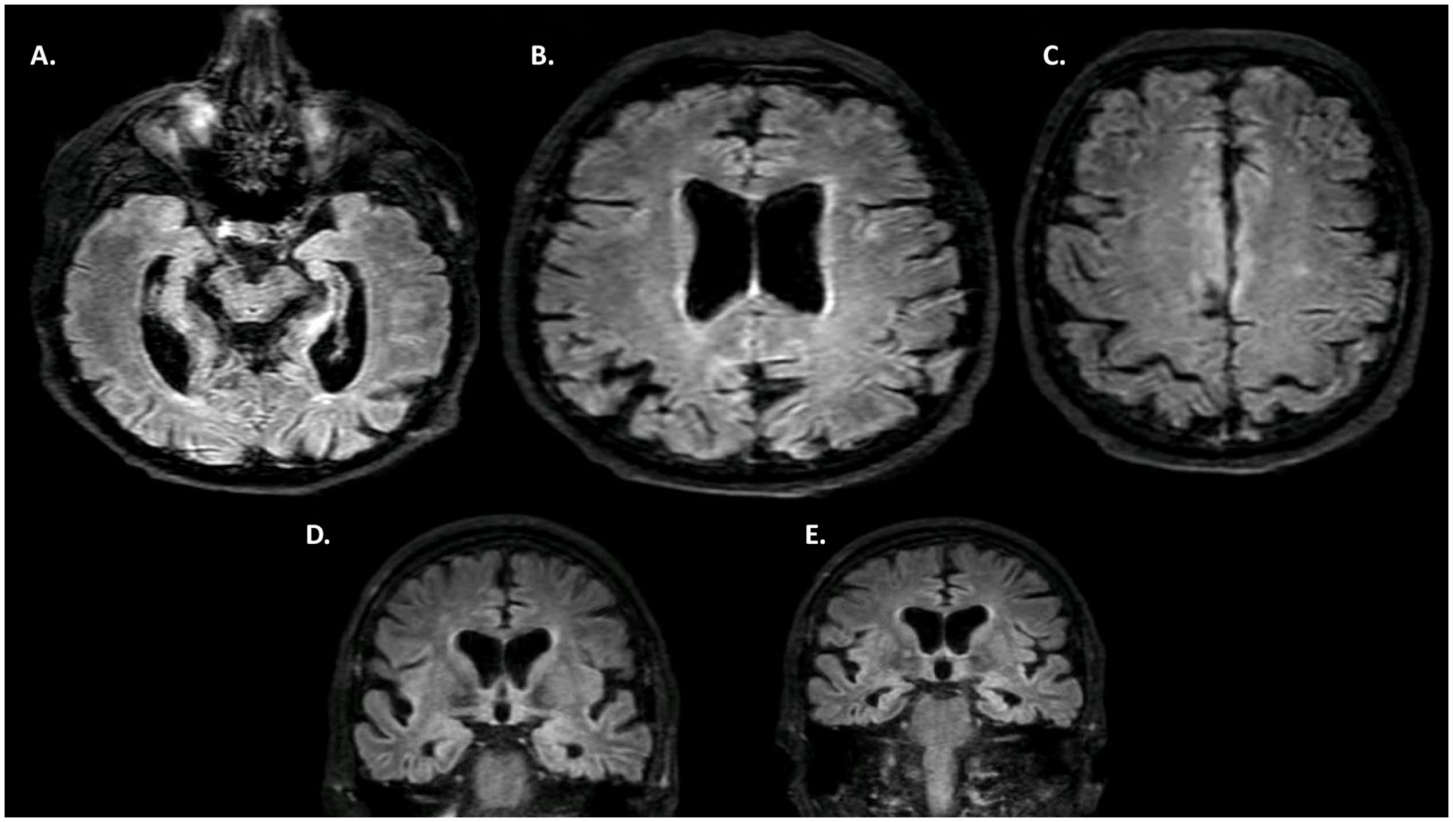
MRI. **(A–C)** FLAIR axial MRI. **(D,E)** FLAIR coronal MRI. Diffuse cerebral atrophy, with a marked gradient in the bi-hippocampal regions (**D,E**—Scheltens 3) and pre-cuneus. Discrete associated vascular leukopathy.

**Figure 2:**
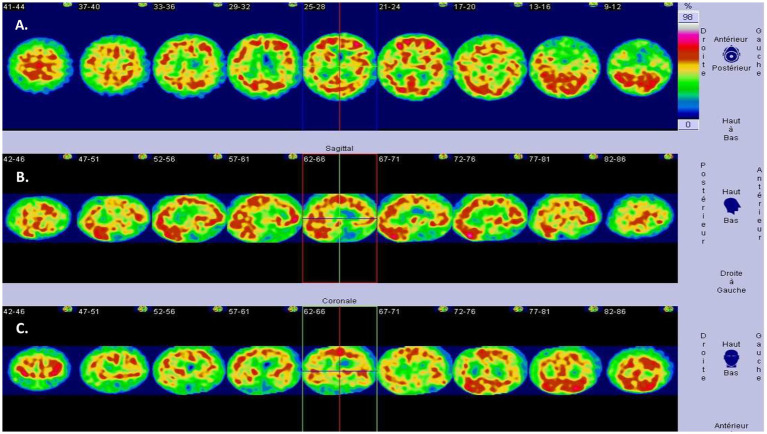
SPECT HMPAO (99mTc) study of regional blood flow. Highly heterogeneous distribution of the cerebral cortex with biparietal and bitemporal hypoperfusion [**(A)** Axial, **(B)** Sagittal, **(C)** Coronal] extending to the posterior frontal regions (low activity in the basal ganglia is also noted).

Blood tests (including complete blood count, metabolic panel, thyroid function, and inflammatory markers) were normal. Brain MRI revealed diffuse atrophy with significant bi-hippocampal atrophy (Scheltens 3/4 on both sides) and parietal lobe atrophy (cuneus—predominating on the right) (Figure 1). Thoraco-abdominal-pelvic CT scan and anti-neuronal antibodies were normal. DaTscan showed bilateral dopaminergic denervation, predominating on the left. HMPAO SPECT scan demonstrated a highly heterogeneous distribution in the cerebral cortex with biparietal and bitemporal hypoperfusion extending to the posterior frontal regions (Figure 2). Additionally, there was low activity in the basal ganglia. EEG showed a non-specific encephalopathy pattern (slow, diffusing toward the anterior regions) with pseudo-periodic graphoelements predominantly in the posterior regions bilaterally. Lumbar puncture did not reveal meningitis. Alzheimer’s disease biomarkers (checked twice in two different laboratories) showed decreased beta-amyloid peptide (265 pg/mL—normal range 562–1,018), Tau protein (112 pg/mL—normal range 116–370), and phospho-Tau (<20 pg/mL—normal range 36–66). The IATI ratio was 0.7 (abnormal, suggesting Alzheimer’s disease clinico-biological profile if less than 0.8). Protein 14-3-3 was negative, as was the intrathecal search for anti-neuronal antibodies. APOE genotype was not performed.

Following the discontinuation of neuroleptics, the patient’s alertness and motor performance improved with physical therapy. The Montreal cognitive Assessment (MoCA) (full neuropsychological tests not feasible) was 18/30.

There were cognitive fluctuations. A major impairment of attentional faculties was noted, along with slowed information processing and working memory deficits. Episodic memory was affected at the retrieval level, while recognition remained preserved. Language was impoverished, with reduced verbal fluency, but overall comprehension was relatively preserved.

**Figure 3:**
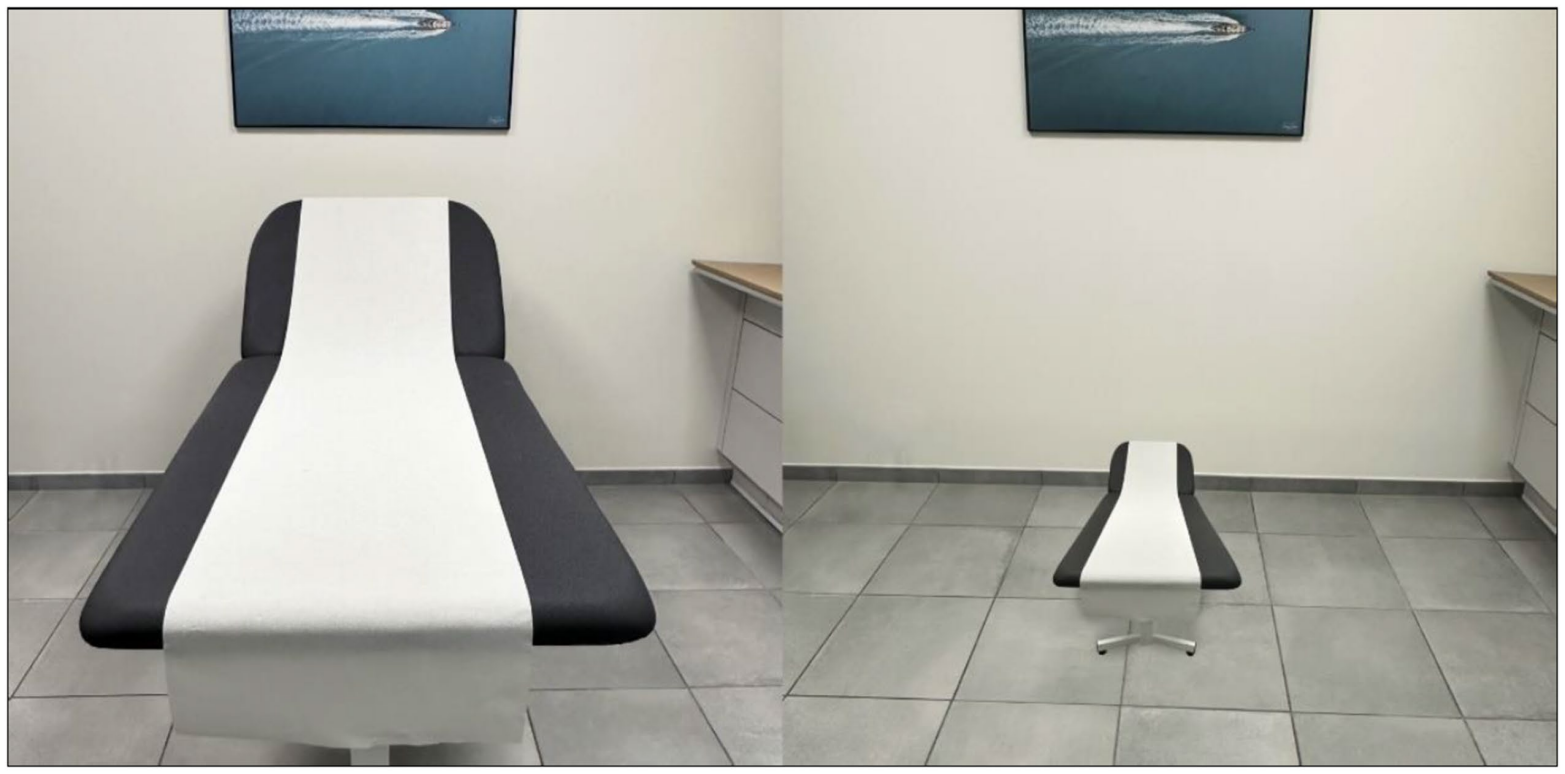
Representation of the visual fore of Alice in Wonderland Syndrome described by the patient (micropsia of an object in the environment of his visual scene) (personal production).

The cognitive domains of memory and visuo-constructive abilities were the most impaired. Tests of visual perception (letters, words, colors, objects, famous faces) and mental imagery were not performed. Visual disturbances were mainly reported, including mixed visual hallucinations (macrozooptic animals, spiders), which were not highly distressing, probable pareidolias, and notably one episode suggestive of a visual form of Alice in Wonderland Syndrome (AIWS): when asked to lie down in his bed, the patient replied fearfully that he could not because “the bed had shrunk” (Figure 3). This was the only episode of its kind documented by the caregivers. Treatment with Keppra was initiated without changes in the symptoms, particularly the visual phenomena. The patient’s general condition rapidly deteriorated, notably with recurrent aspiration pneumonia complicating axial symptoms, leading to the patient’s death 2 months after hospitalization.

## Discussion

This case represents the first description of AIWS in a patient with dementia with Lewy bodies (DLB) associated with Alzheimer’s pathology. The clinical presentation—marked by progressive cognitive decline, parkinsonism, and fluctuating visual hallucinations—is consistent with the limbic-early subtype of LBD, which frequently exhibits concomitant Alzheimer’s disease pathology (12, 15, 16). Neuroimaging and cerebrospinal fluid biomarkers further corroborate the dual proteinopathy.

Our case raises important considerations regarding the pathophysiology of visual perception in neurodegeneration (17–20). The transient report of an abnormally “shrinking” bed suggests a disruption in high-level visual cognition, specifically the integration of canonical knowledge regarding object size. We hypothesize that dysfunction in the right extrastriate cortex and associated white matter tracts-areas critical for processing and storing canonical object properties—may underlie this phenomenon. Recent studies have localized such dysfunction to the V4 area and the ventral occipital fasciculus (21, 22).

It is essential to note that the patient’s brief AIWS episode occurred in the context of multiple neuroleptic administrations. Although antipsychotics may bias clinical presentation, the singularity and rapid resolution of the AIWS symptom argue against a primary medication effect. Furthermore, the exclusion of schizophrenia spectrum disorders (with careful clinical review revealing no additional psychotic symptoms) strengthens the attribution of AIWS to the underlying neurodegenerative process (23–26).

Additionally, the presence of significant hypoperfusion on HMPAO SPECT aligns with the observed memory deficits. While vascular damage is often associated with affective lability, no abrupt mood changes were noted in our patient. Rather, the hypoperfusion appears to contribute to memory loss within the broader neurodegenerative framework.

Finally, the integration of our findings with contemporary theories of visual cognition is intriguing (27–29). The classical model of visual processing—entailing bottom-up acquisition of sensory data followed by top-down interpretation—underscores the malleability of object size perception (30). We propose that the agnosia of object size observed in this case reflects a selective failure of the high-level associative network that compares incoming visual information with stored canonical object properties. This dysfunction may be conceptualized within a new paradigm of TBI-associated proteinopathies and neurodegenerative disruption, paralleling insights from studies on chronic traumatic encephalopathy. Our discussion is enriched by drawing parallels with the work of Husserl on the “primordial body” (Urleib) and intuition, as well as the transformative interpretations of visual perception found in surrealist art.

## Conclusion

**Figure 4:**
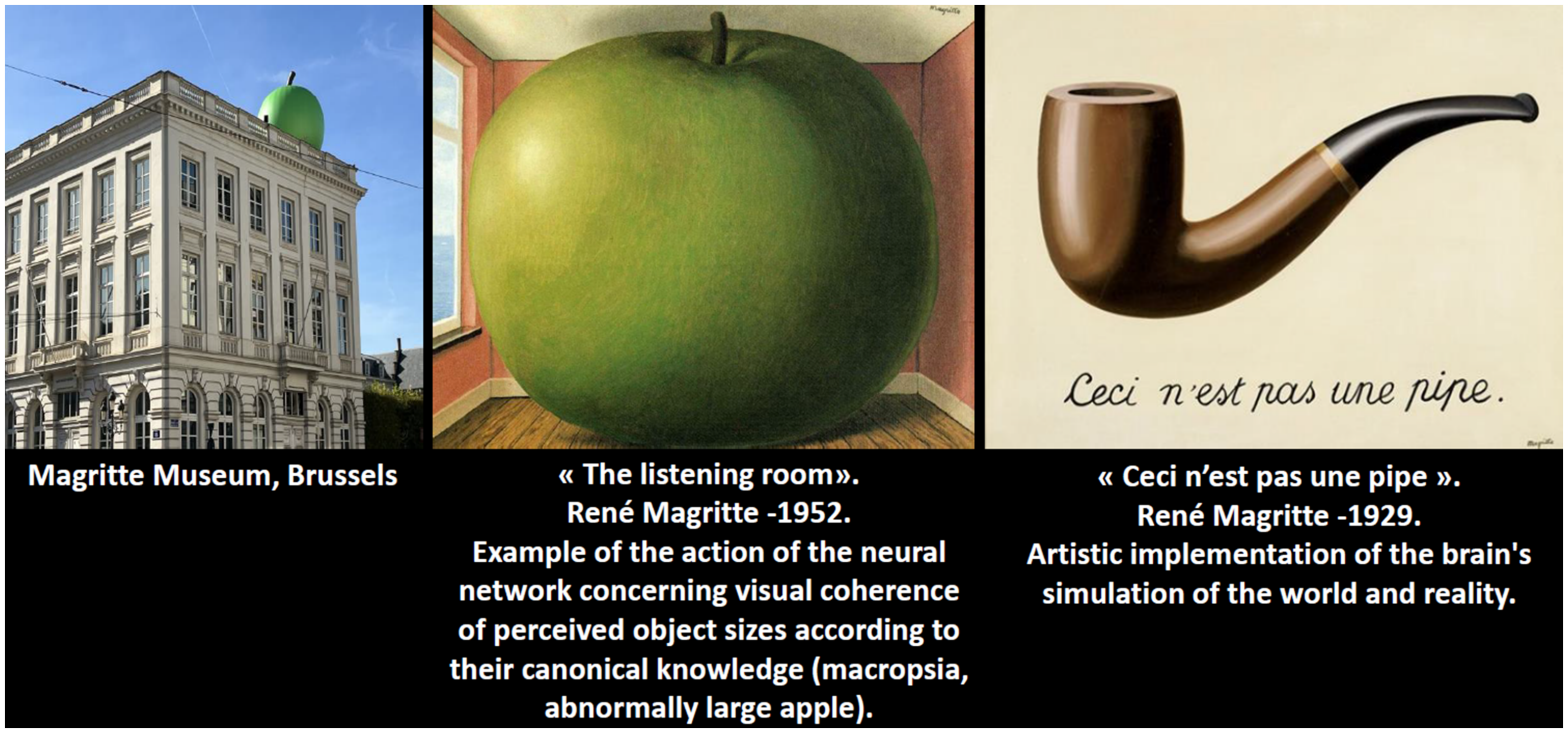
Magritte’s art transforms reality, reflecting his imagined world and illustrating the surrealist idea that our perception differs from actual vision. He understood this concept intuitively, anticipating discoveries in modern neuroscience.

This case report is the first to document an episode of Alice in Wonderland Syndrome in a patient with dementia with Lewy bodies. Our findings suggest that selective dysfunction in high-level visual networks—responsible for integrating canonical object size information—may lead to an agnosia of object size. Although the patient’s neuroleptic treatment may have introduced some clinical bias, the overall evidence supports a neurodegenerative etiology rather than a primary psychiatric disorder. Future studies should aim to delineate biomarkers (e.g., age at onset, genetic predisposition, severity of initial deficits) that can stratify patients with similar proteinopathies. Moreover, further exploration of the parallels between neurodegenerative visual disturbances and the intuitions expressed in surrealist artwork may enrich our understanding of the complex interplay between perception and reality (Figure 4).

## Data Availability

The original contributions presented in the study are included in the article/supplementary material, further inquiries can be directed to the corresponding author.
